# Long-Term Outcome of Immunosuppression in a Patient With Immunoglobulin A Nephropathy and Serum-Positive Antineutrophil Cytoplasmic Antibodies

**DOI:** 10.7759/cureus.110839

**Published:** 2026-06-14

**Authors:** Chenuka S Wijayasinghe, Martin Klein, Muhammad M Javaid

**Affiliations:** 1 Medicine, South West Healthcare, Warrnambool, AUS; 2 School of Medicine, Deakin University, Warrnambool, AUS; 3 Rural Health, Monash University, Mildura, AUS

**Keywords:** anca, antineutrophil cytoplasmic antibodies, iga nephropathy, immunoglobulin a nephropathy, vasculitis

## Abstract

The concurrent occurrence of immunoglobulin A nephropathy (IgAN) and serum-positive antineutrophil cytoplasmic antibodies (ANCA) is a rare clinical entity frequently associated with more severe disease progression. Without appropriate intervention, affected individuals may rapidly advance to end-stage kidney disease (ESKD), necessitating renal replacement therapy. Early initiation of intensive induction immunosuppressive therapy has the potential to enhance renal outcomes, which can be maintained over time with suitable maintenance regimens. In this report, we describe the management of a patient with pre-existing chronic kidney disease (CKD) and impaired baseline renal function who was treated with cyclophosphamide for induction and azathioprine for maintenance for crescentic IgAN and serum-positive ANCA. This approach resulted in substantial improvement in renal function, which was preserved over a 55-month follow-up period.

## Introduction

Immunoglobulin A nephropathy (IgAN) is among the most frequently encountered glomerular diseases in clinical practice, typically presenting as chronic, slowly progressive kidney disease. A minority of patients (less than 10%) develop rapidly progressive disease, often showing extracapillary proliferation and crescent formation on kidney biopsy. These findings indicate severe inflammation, with immune cells and fibrin accumulating in Bowman's space [1]. The co-occurrence of IgAN with serum-positive antineutrophil cytoplasmic antibodies (ANCA) is relatively rare, constituting less than 3% of all IgAN cases. The simultaneous presence of crescentic proliferation in individuals with both IgAN and ANCA positivity is exceedingly uncommon, reported in only 0.14% of IgAN patients in recent studies [1,2]. Optimal management strategies for these patients, who frequently present with features indicative of rapidly progressive glomerulonephritis (RPGN), have yet to be determined, and it remains uncertain whether this combination represents an overlap syndrome or a distinct pathological entity [3]. Several case reports have documented favorable responses to immunosuppressive therapy in this cohort, suggesting that this distinction is of limited clinical significance and does not influence management strategies [3-5]. Nonetheless, long-term outcomes associated with such aggressive treatment, particularly in those with significant pre-existing chronic kidney disease (CKD), remain inadequately characterized. We present the case of a patient with IgAN and ANCA-associated vasculitis (AAV) overlap syndrome who was treated with induction and maintenance immunosuppressive therapy, with 55 months of follow-up demonstrating a favorable long-term outcome with this management strategy.

The initial results of the case were previously presented as a poster at the 2022 ANZSN Annual Scientific Meeting on April 17-19, 2022.

## Case presentation

A 64-year-old female presented to a regional hospital with a one-week history of worsening peripheral edema. Her medical history was significant for type two diabetes mellitus, hypertension, sarcoidosis with granulomatous liver involvement, monoclonal gammopathy of undetermined significance (MGUS), and CKD, with a baseline serum creatinine of 1.24 mg/dL and an estimated glomerular filtration rate (eGFR) of 45 ml/min/1.73m². Her regular medications included metformin, irbesartan, hydrochlorothiazide, quetiapine, tranylcypromine, ranitidine, and vitamin D.

On admission, initial investigations revealed marked deterioration in renal function, with a serum creatinine of 3.35 mg/dL and an eGFR of 14 ml/min/1.73 m². Urinalysis demonstrated a urine albumin-creatinine ratio (uACR) of 3363 mg/g and significant microhematuria, with 660 red blood cells per high-power field (RBC/HPF) on urine microscopy. Inflammatory markers were elevated: C-reactive protein (CRP) measured 21 mg/L, and erythrocyte sedimentation rate (ESR) was 107 mm/hr. Renal tract ultrasound was unremarkable.

Due to acute deterioration in renal function, her metformin, irbesartan, and hydrochlorothiazide were discontinued. Furosemide was started to manage fluid overload. Owing to the patient's clinical presentation, a primary glomerular pathology was suspected. Accordingly, a comprehensive glomerulonephritis screen was undertaken, including ANCA, antinuclear antibodies (ANA), anti-double-stranded DNA (dsDNA) antibodies, anti-glomerular basement membrane (GBM) antibodies, and complement components C3 and C4. The patient was subsequently referred to a tertiary renal unit for a kidney biopsy to establish a definitive diagnosis. While awaiting transfer for biopsy, she received intravenous methylprednisolone 500 mg daily for three consecutive days. During this interval, renal function continued to deteriorate, with serum creatinine increasing to 4.57 mg/dL and eGFR declining to 10 ml/min/1.73 m².

**Figure 1:**
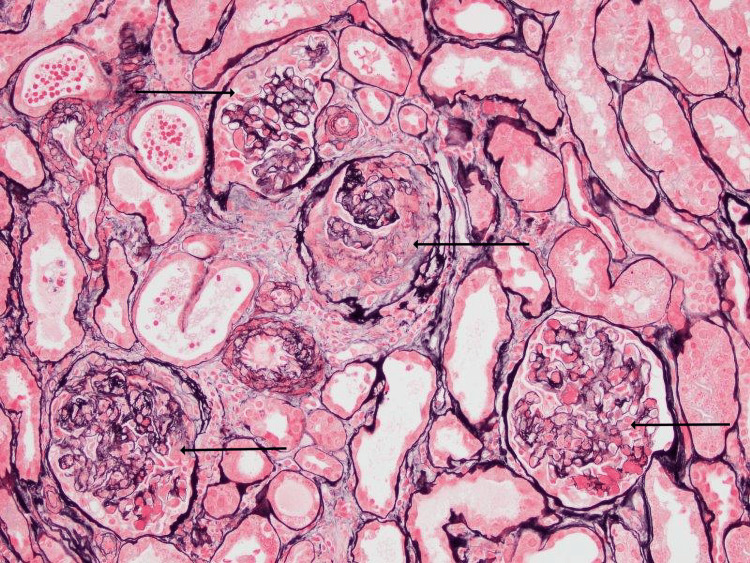
Light microscopy demonstrates mesangial matrix expansion, heightened mesangial and endocapillary cellularity, and the presence of fibrocellular crescents

Renal biopsy was performed four days after admission, which revealed 16 glomeruli, including one globally sclerosed and six exhibiting segmental sclerosis. There was diffuse mesangial matrix expansion and increased mesangial and endocapillary cellularity; six glomeruli had fibrocellular or cellular crescents (Figure 1). One glomerulus showed segmental fibrinoid necrosis. The biopsy also demonstrated moderate interstitial fibrosis and tubular atrophy, with patchy mild chronic inflammation.

**Figure 2:**
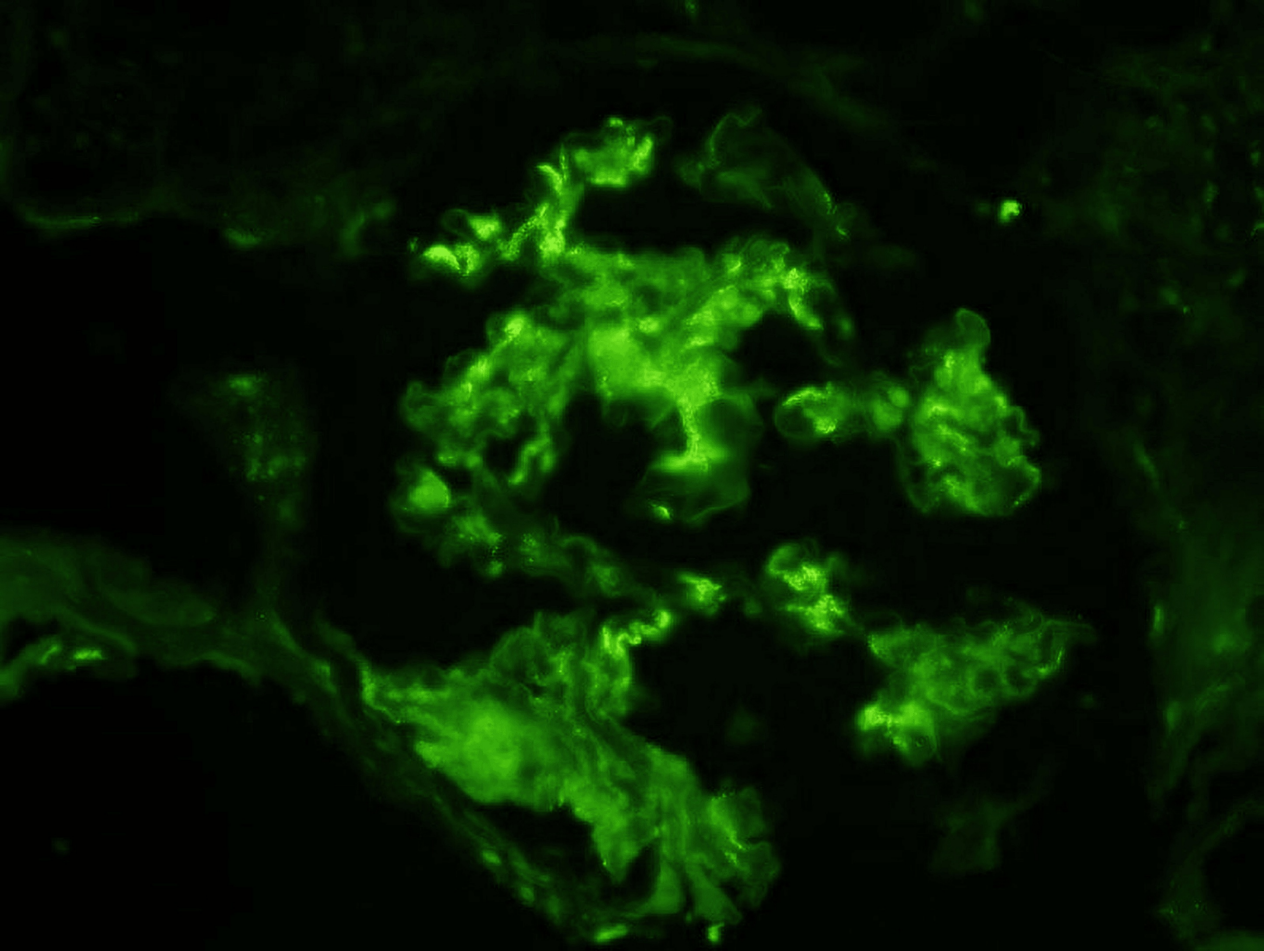
Immunofluorescence microscopy demonstrates granular mesangial and capillary immunoglobulin A staining

Immunofluorescence showed granular mesangial and capillary staining for IgA and C3, with weak positivity for C1q, IgG, IgM, kappa, and lambda (Figure 2). Fibrin and albumin stains were negative. The findings led to a diagnosis of IgAN, classified as M1, E1, S1, T1, C2 according to the Oxford criteria.

Prednisolone was commenced at 80 mg daily, and the patient was discharged with arrangements for outpatient nephrology follow-up at the regional hospital. At review four weeks after discharge, renal function remained significantly impaired, with a serum creatinine of 4.7 mg/dL and an eGFR of 9 ml/min/1.73 m². Nephrotic-range proteinuria persisted (uACR 3522 mg/g), accompanied by worsening hypoalbuminemia, with serum albumin decreasing from 31 g/L to 26 g/L. Serological results from the initial admission became available at this time and demonstrated positive ANCA with an anti-myeloperoxidase (MPO) titer of 7.8 AI. ANA, anti-dsDNA antibodies, anti-GBM antibodies, and complement C3 and C4 levels were within normal limits. Screening for hepatitis B, hepatitis C, and HIV was negative. Serum angiotensin-converting enzyme was elevated at 75 U/L, consistent with the patient's known sarcoidosis. Serum protein electrophoresis demonstrated a monoclonal IgA kappa band, in keeping with her history of MGUS.

In view of the suboptimal response to corticosteroid monotherapy, positive serum ANCA, and histopathological features overlapping with ANCA-associated glomerulonephritis, including crescents and fibrinoid necrosis on kidney biopsy, the possibility of dual pathology comprising IgAN and AAV was considered. The patient was therefore commenced on monthly intravenous cyclophosphamide (1 g, 0.5 g/m²) for six months. Two weeks after the first dose, her white cell count (WCC) dropped to 4.9 × 10^9/L from a previous level of 17.1 × 10^9/L. Cyclophosphamide-induced myelosuppression was suspected, and the dosage was reduced by 25% for subsequent infusions to prevent further reduction in WCC and to avoid the increased risk of infection. WCC remained stable and above 5.0 × 10^9/L following subsequent infusions, and no further dose reduction was required. Renal function showed improvement after the initiation of cyclophosphamide therapy. Following the first dose, the patient's serum creatinine decreased to 3.52 mg/dL, corresponding to an eGFR of 13 ml/min/1.73m². Continued treatment resulted in further improvement of kidney function.

**Figure 3:**
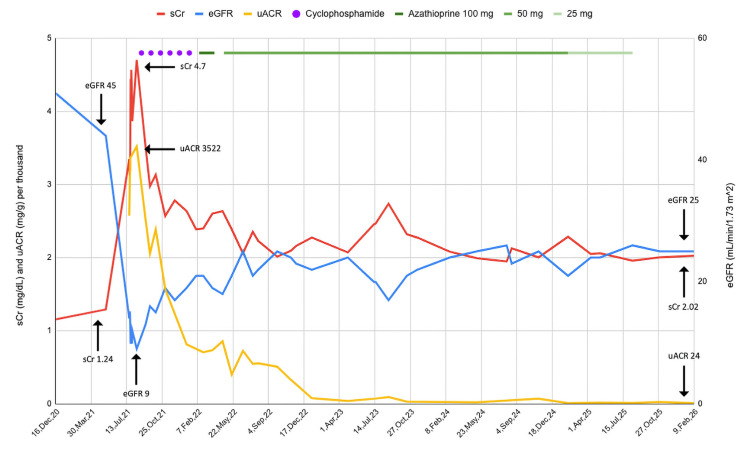
Illustration depicting the patient's clinical progression throughout the 55-month follow-up interval eGFR - estimated glomerular filtration rate; SCr - serum creatinine; uACR - urine albumin-creatinine ratio

At six months, creatinine measured 2.39 mg/dL, eGFR was 21 ml/min/1.73m², uACR was 704 mg/g, and ANCA serology was negative. Prednisolone was gradually tapered to a maintenance dose of 5 mg daily over six months. Following completion of cyclophosphamide induction therapy, low-dose azathioprine (1 mg/kg) was initiated one month later as maintenance therapy. After two months, the dosage was halved in response to a decline in WCC to 2.8 × 10^9/L, which subsequently improved to normal limits. Renal function stabilized at an eGFR of 25 ml/min/1.73 m². Proteinuria demonstrated ongoing improvement, with uACR decreasing to 27 mg/g after twelve months of therapy (Figure 3). Due to continued clinical stability, azathioprine therapy was discontinued after approximately fifty months, with close monitoring implemented thereafter. Six months after cessation of immunosuppression, the patient stayed in remission and maintained stable parameters, including a creatinine level of 2.02 mg/dL, an eGFR of 25 ml/min/1.73 m², and a uACR of 24 mg/g (Figure 3). Serum ANCA remained negative.

## Discussion

A positive serum ANCA result does not definitively indicate AAV, as it may occur in other conditions as well. Similarly, renal immunoglobulin A (IgA) deposits have been observed in the general population without evidence of renal disease [6]. A few studies suggest that ANCA positivity in patients with IgAN is coincidental and lacks clinical significance, particularly in cases with slow disease progression and no crescents on biopsy [7]. In contrast, other reports propose that ANCA-positive IgAN constitutes a distinct entity characterized by unique features. Increasing evidence supports the view that this combination represents a unique clinical entity with its own specific clinical characteristics [1,3,8].

Yang et al. found that ANCA-positive IgAN patients exhibit clinical features similar to those of AAV, including hematuria, nephritic syndrome, and RPGN. Additionally, systemic manifestations and pulmonary involvement were observed more frequently in this cohort and were comparable to those of AAV patients. Compared with ANCA-negative IgAN, ANCA-positive IgAN patients had a higher percentage of glomerular fibrinoid necrosis and responded better to immunosuppressive therapy, with a higher likelihood of dialysis withdrawal and avoidance of end-stage kidney disease (ESKD) within six months [8]. Therefore, consistent assessment of ANCA levels and the application of effective immunosuppressive therapies are essential strategies to enhance long-term renal outcomes in these patients.

Bantis et al. reported on eight ANCA-positive IgAN patients and compared them with 26 ANCA-negative IgAN patients with more than 10% crescentic glomeruli. All ANCA-positive patients developed RPGN, whereas only one-third of ANCA-negative patients presented with RPGN. ANCA-positive patients reached a higher peak serum creatinine level within the first three months (4.2 ± 2.2 vs 2.5 ± 1.9 mg/dL; eGFR, 19.3 ± 10.2 vs 45.9 ± 30.1 ml/min/1.73 m²). Furthermore, the ANCA-positive group had a higher proportion of crescentic glomeruli (54.3% ± 18% vs 34.5% ± 26%), and all patients showed dominant IgA staining in the mesangium. IgG was positive in three patients; C3, in six patients; C1q, in five patients; and IgM and fibrin in all eight patients. All ANCA-positive IgAN patients were treated with a combination of cyclophosphamide and corticosteroids. Similar to our case, four patients received monthly intravenous cyclophosphamide at 0.5 g/m², and the other four were treated with oral cyclophosphamide at 2mg/kg daily for six months. Maintenance therapy comprised cyclophosphamide in two patients, azathioprine in four, and mycophenolate mofetil in two patients. After cyclophosphamide and steroid treatment, kidney function improved in all ANCA-positive patients, and ANCA became negative. In contrast, none of the ANCA-negative patients experienced renal recovery under the same regimen [3].

Another case series reported four patients with ANCA-positive crescentic IgAN. All patients presented with nephritic syndrome, comprising microscopic hematuria, proteinuria, RPGN, and hypertension. All patients but one had eGFR below 15 ml/min/1.73m² at diagnosis. Kidney biopsies showed mesangial proliferation with dominant IgA deposits and variable IgG and C3 staining on immunofluorescence. Cellular and fibrocellular crescent formation was observed in all cases, affecting 17%-75% of the glomeruli. All patients received corticosteroids and intravenous cyclophosphamide at 500 mg/m² every four weeks for six months. Azathioprine at 2 mg/kg was used as maintenance therapy for at least 12 months. All but one patient who required initiation of dialysis at five months due to intractable extracellular volume overload responded to the induction therapy. One patient had a renal-limited relapse after five years, which was managed using the same induction therapy. However, the patient died at three months due to infection [6].

More recently, Kaseda et al. reported their experience of managing an 18-year-old patient who presented with unexplained macroscopic hematuria, abdominal pain, fever, proteinuria measuring 2.0 g/g creatinine, and renal impairment with serum creatinine of 2.6 mg/dL and eGFR of 22.3 ml/min/1.73 m². Serum ANCA was positive. Kidney biopsy showed mild to moderate mesangial hypercellularity, endocapillary hypercellularity, and fibrinoid necrosis. Cellular and fibrocellular crescents were observed in 60% of glomeruli. Immunofluorescence showed granular deposits of moderate IgA and weak C3. The biopsy findings led to the diagnosis of IgAN. Patient was treated with four weekly doses of 500 mg rituximab and 40 mg daily of oral prednisolone for 28 days, followed by avocapan at 60 mg daily. Thirteen months post-treatment, the patient demonstrated normal renal function, negative ANCA, absence of hematuria, and minimal proteinuria [4].

While definitive conclusions cannot be established from a single case, our findings highlight the potential benefit of aggressive immunosuppressive therapy in patients with IgAN and ANCA-positive status for preserving renal function and delaying the onset of ESKD. The patient exhibited severe renal impairment that did not respond to high-dose corticosteroid monotherapy for more than four weeks. Despite adverse prognostic factors, including poor baseline renal function, a history of diabetes mellitus, and chronic changes on kidney biopsy, the patient responded favorably to cyclophosphamide induction therapy. This response was maintained for nearly five years with azathioprine maintenance treatment.

## Conclusions

The coexistence of crescentic IgAN on kidney biopsy and positive serum ANCA may represent a distinct clinicopathological entity, which frequently presents as RPGN. In such cases, early recognition is important, as prompt initiation of intensive immunosuppressive therapy may help preserve renal function and delay progression to ESKD requiring kidney replacement therapy. This potential benefit may extend even to patients with pre-existing CKD, markedly reduced kidney function at presentation, or superimposed chronic changes on kidney biopsy.

## References

[1] Agraz I, Castañeda Z, Sanz-Martínez MT (2022). The presence of ANCA in IgA crescentic nephropathy does not lead to worse prognosis with intensive rescue treatment. J Clin Med.

[2] Li W, Chen R, Chen W, Huang F, Xia X (2023). Clinicopathological features and outcomes of IgA nephropathy with serum antineutrophil cytoplasmic autoantibody positivity. Am J Nephrol.

[3] Bantis C, Stangou M, Schlaugat C (2010). Is presence of ANCA in crescentic IgA nephropathy a coincidence or novel clinical entity? A case series. Am J Kidney Dis.

[4] Kaseda K, Terakawa R, Matsui R (2025). Successful treatment of MPO-ANCA positive crescentic IgA nephropathy/IgA vasculitis with nephritis potentially triggered by a COVID-19 vaccine in a young adult female using corticosteroids, rituximab, and avacopan. CEN Case Rep.

[5] Tan SL, Potezny T, Li JY (2022). The successful use of rituximab in crescentic IgA nephropathy with concurrent ANCA positivity. Nephrology (Carlton).

[6] Ștefan G, Terinte-Balcan G, Stancu S, Zugravu A, Gherghiceanu M, Mircescu G (2021). IgA nephropathy with serum ANCA positivity: case series and literature review. Rheumatol Int.

[7] Koratala A, Zeng X, Kazory A (2017). ANCA-positive IgA nephropathy without necrotising or crescentic glomerulonephritis: a clinical conundrum. BMJ Case Rep.

[8] Yang YZ, Shi SF, Chen YQ (2015). Clinical features of IgA nephropathy with serum ANCA positivity: a retrospective case-control study. Clin Kidney J.

